# The Work of Nurses in Primary Health Care: Crossings of the New Public Management

**DOI:** 10.3390/healthcare11111562

**Published:** 2023-05-26

**Authors:** Maristel Silva Kasper, Felipe Lima dos Santos, Poliana Silva de Oliveira, Janaina Pereira da Silva, Karen da Silva Santos, Priscila Norié de Araujo, Gabriella Carrijo Souza, Cássia Bianca de Souza Quintão, Angelina Lettiere Viana, Silvia Matumoto, Silvana Martins Mishima, Tauani Zampieri Fermino, Ana Lucia Abrahão, Liane Beatriz Righi, Gilles Monceau, Cinira Magali Fortuna

**Affiliations:** 1 Public Health Nursing Graduate Program, University of São Paulo at Ribeirão Preto College of Nursing, Ribeirão Preto 14040-902, São Paulo, Brazil; maristelkasper@gmail.com (M.S.K.); fel1pesantos@yahoo.com.br (F.L.d.S.); polianasilva@usp.br (P.S.d.O.); janaina.pereira.silva@usp.br (J.P.d.S.); karen.santos@usp.br (K.d.S.S.); priscila.araujo@usp.br (P.N.d.A.); gabriella.souza@usp.br (G.C.S.); 2 Laboratoire École-Mutations-Apprentissages, CY Cergy Paris Université, 92230 Gennevilliers, France; gilles.monceau@cyu.fr; 3 Laboratoire Interdisciplinaire de Recherche sur les Transformations des Pratiques Educatives et des Pratiques Sociales, Université Paris-Est-Créteil-Val-de-Marne, 94010 Créteil, France; 4 Laboratoire Éducation et Diversité en Espaces Francophones, 87036 Limoges, France; 5 Technology and Innovation in Nursing Professional Master Program, University of São Paulo at Ribeirão Preto College of Nursing, Ribeirão Preto 14040-902, São Paulo, Brazil; cassia.quintao@pbh.gov.br; 6 Public Health Nursing Graduate Program, Department of Maternal-Infant and Public Health Nursing, University of São Paulo at Ribeirão Preto College of Nursing, Ribeirão Preto 14040-902, São Paulo, Brazil; angelina.lettiere@usp.br (A.L.V.); smatumoto@eerp.usp.br (S.M.); smishima@eerp.usp.br (S.M.M.); tauanizampi@usp.br (T.Z.F.); 7 Department of Medical-Surgical Nursing, Aurora de Afonso Costa College of Nursing at Fluminense Federal University, Niterói 24020-090, Rio de Janeiro, Brazil; abrahaolucia@hotmail.com; 8 Department of Collective Health, Health Sciences Center of the Federal University of Santa Maria, Santa Maria 97105-900, Rio Grande do Sul, Brazil; lianerighi@gmail.com

**Keywords:** work, nurses, primary care nursing, primary health care, health management

## Abstract

The literature in the field of health management mentions a concept called new public management (NPM), introduced in Brazil and France at the end of the 20th century. The objective of the study was to analyze the repercussions of the work of nurses in primary health care in Brazil and France under the influence of NPM. This is an excerpt of a double-titled thesis, which is a research intervention with nurses from two Brazilian states and five French departments. Data were produced between February 2019 and July 2021. The public policy Health on the Hour acted as an institutional transducer, provoking a reduction in access and producing effects on professional practices. In both countries, NPM amplified the predominance of technical and quantifiable acts, the focus on individual care, and the loss of autonomy. Nurses reported insurmountable situations, using the metaphor “Sophie’s choice”. The results showed that making dilemmatic decisions has been the daily routine of nurses, which has not resulted in debureaucratization and higher quality of care.

## 1. Introduction

The literature in the field of health management mentions a new concept called New Public Management (NPM), introduced in Brazil and France at the end of the 20th century. The model incorporates new concepts in public management, such as flexibility, accountability, reform, modernization, fiscal balance, strategic management, quality improvement, effectiveness, increased efficiency, definition of strategic objectives, definition of indicators and goals, contractualization of results, evaluation of institutional performance and team productivity, and rationalization of spending, among others [1,2,3].

NPM originated in 1975 in England and in countries with British influence, such as Australia and New Zealand, as an alternative to bureaucratic public administration, proposing to increase the quality of services and reduce costs. The term NPM was first mentioned in the work *La Nouvelle Gestion Publique: pour un État sans Bureaucratie*, by Michel Messenet and Octave Gélinier, 1975 [2]. The term came into wider use with Christopher Hood’s (1991) work *A public management for all seasons?* [2]. In France and Brazil today, we speak of NPM as the new mode of public management that has been systematized since the early 2000s [3,4].

NPM has been implemented in different ways around the world, including within health systems. Brazil stands out with the constitution of a universal health system in a diverse set of formats, most of which are of the social insurance type [5]. France has a health system classified as health insurance type, working with co-payment systems similar to the social insurance systems present in most Latin countries [6].

In Brazil, three studies have analyzed the effects of managerialism on the professional practices of physicians, one in a hospital and two others in primary health care (PHC) [6,7,8,9]. In France, there are studies on the reorganization of professions and on performance indicators [10,11]. However, there are no studies specifically on nursing in the context of PHC. Most of the studies in French and international literature on the effects of rationalization in health care focus on the medical profession, and less so on other health care professions [12].

Several studies that have analyzed the impact of NPM on nursing practices point to transformations in the hospital setting. However, none of the studies identified in the scientific literature analyzed this impact in the PHC field in either Brazil or France [13,14,15,16,17,18,19,20,21,22,23,24,25,26,27]. A study conducted in Portugal on nurses, physicians, and clinical secretaries investigated the transformations in the management of family health units (FHU), showing that 84.2% of professionals reported a moderate to high level of occupational stress, with nurses having the highest scores [28]. Therefore, in view of the national and international evidence on the institutionalization of NPM in the health field, especially in the hospital sector, the present double-titled doctoral research was developed with the purpose of understanding how management models impact the professional practices of PHC nurses in Brazil and France.

In 2019, the Brazilian federal government created new health policies that were considered the “bases of the PHC reform”, among them being the Previne Brasil Program, Health on the Hours (Saúde na Hora), and the Doctors for Brazil Program (Médicos pelo Brasil). The Health on the Hour (Saúde na Hora) Program’s mission was to expand access by extending the opening hours of primary care units [29].

Therefore, the aim of this study was to analyze the repercussions of nurses’ work in primary health care in Brazil and in primary care in France, under the influence of new public management.

## 2. Materials and Methods

### 2.1. Study Design

This is a qualitative, exploratory study from the perspective of the theoretical and methodological framework of Institutional Analysis, socio-clinical line [30,31,32,33]. The framework offers instruments to analyze the evolution of nurses’ work in PHC via analysis of the institutions that permeate the professional practices. In this type of research, the objectives are localized to institutional processes, the analysis of which requires a sensitive approach to institutional contradictions [31].

### 2.2. Theoretical-Methodological Framework

The institutional socio-clinical framework proposes eight principles to be observed in studies of this type: analysis of the order and demands, participation of the subjects in the study, the work of the analyzers, analysis of transformations that occur as the work progresses, the application of the modalities of restitution, the analysis of the primary and secondary implications, the intention of the production of knowledge, and attention to context and institutional interferences [32]. The study followed the list of criteria included in the Consolidated Criteria for Reporting Qualitative Research (COREQ) [34].

### 2.3. Study Setting

The study was carried out in the south and southeast of Brazil. It took place in Ribeirão Preto, a city located in the interior of the state of São Paulo, and Porto Alegre, capital of the state of Rio Grande do Sul, and in five departments in France: Bouches-du-Rhône, Essone, Val d’Oise, Yvelines, and Maine et Loire. The choice of these places was intentional because they presented management models that referred to NPM and were located in different regions of the two countries.

### 2.4. Participants

In Brazil, nurses from family health teams (ESF) from family health centers (NSF) in Ribeirão Preto, state of São Paulo, and from basic health units (UBS), ESF, and the street clinic in Porto Alegre, state of Rio Grande do Sul, participated in the study. In France, nurses from the Centre Municipal de Santé, the Maison de Santé, the Polo de Santé, the Centre for Family Planning and Education (CPEF), the Centre for Maternal and Child Protection (PMI), the Centre Médico-Psicologique (CMP), the Home Nursing Service (SSIAD), private nursing practice, the crèche, the college, and the lycée took part in the study.

### 2.5. Sampling, Sample Size and Non-Participation

The participants were selected by convenience sampling, being identified via referral by the nurses in charge of training in France, or by nurses who worked in health services and had a network of contacts with PHC nurses in Brazil and primary care nurses in France. The study participants were 30 nurses: 15 Brazilian and 15 French. The period of data collection occurred between February 2019 and July 2021, performed intermittently in both countries. The study data collection time was two (2) years due to the fact that it took place in two countries, Brazil and France, and in different regions in each country. The COVID-19 pandemic also interfered, as there were impediments to displacement and overload of health professionals who prioritized patient assistance to the detriment of participation in this study. Among the inclusion and exclusion criteria, we included nurses who had been working in PHC for at least one year, and excluded those on maternity leave and sick leave.

To minimize the risk of sampling bias in the selection of study participants, socio-clinical research uses the concept of implication analysis, in which the researcher analyzes their ideological, libidinal and organizational implications in addition to their beliefs, personal and professional values in order to broaden their field of vision in the context of the study and visualize important aspects [31].

Numerous refusals to participate were received, especially in the French context at the height of the pandemic. Among the reported causes, nurses claimed: “I’m sorry, but I can’t right now”, and, “Unfortunately I’m completely overloaded”.

### 2.6. Research Instruments, Collecting and Organizing Data

The sources and research instruments used were document analysis, the research diary, individual interviews, and restitution. In this publication, we have chosen to present the results from the interviews. First contact with the participants was made in person at the health service destinations or by telephone. Those who accepted the invitation were invited to schedule an interview date. The interviews were scheduled according to the availability of the participants. The audio was recorded and later transcribed (in full) for analysis. In the case of the French interviews, we chose to use the Sonix software free online version (Sonix Inc., Zhubei City, Taiwan, 2021) for transcription.

Most of the interviews took place face-to-face at the participants’ place of work. However, during the lockdown periods caused by the COVID-19 pandemic, seven interviews were conducted remotely via Google Meet or Zoom. They lasted an average of 90 min and followed a guide consisting of the following themes: professional practices in PHC, work process in PHC, difficulties and/or facilities in working in PHC, and production of care versus production of procedures.

### 2.7. Data Analysis

Cross-questioning of practices was the methodological approach used, which aims to value the particularity and uniqueness of the cases, not only what is universal in both. In other words, this method highlights the elements of one case that illuminate elements in the other case, and allow a better understanding of each one through a mirroring of the practices [35]. This type of methodological approach favors the study of the effects of macro-social issues on a profession with the same name, but a different history, culture, formation, and roles. As for the organization of the data, we opted for the French-Canadian proposition, which consists of three phases: transcription, transposition and reconstitution [36]. For the data analysis, we followed the eight principles of institutional socio-clinics [30,31,32,33].

### 2.8. Ethical Aspects

The study was conducted according to the guidelines of the Declaration of Helsinki, and approved by the Research Ethics Committee of University of São Paulo at Ribeirão Preto College of Nursing under Certificate of Presentation for Ethical Consideration number 03164018.8.0000.539 and Report number 3.134.647, issued on 6 February 2019, and also approved by the Research Ethics Committee of Health Secretariat of Porto Alegre-RS under Certificate of Presentation for Ethical Consideration number 03164018.8.3001.5338 and Report number 3.267.686, issued on 16 March 2019 in accordance with the Guidelines and Regulatory Standards for Research with Human Subjects, Resolution number 466/2012 of the National Health Council of the Brazilian Ministry of Health. In France, no ethics committee approval was required, but we followed the Brazilian regulations and presented the informed consent form (ICF) to the French participants, explaining the objectives, risks, and benefits of research participation. All participants signed an ICF.

In order to guarantee the anonymity of the research participants, in the presentation of the verbatims, the letter “N” was used to indicate nurse study participants, followed by a sequential number from one to twenty-eight, according to the order of the interviews, and followed by the abbreviation of the name of the country: BRA for Brazil and FRA for France.

## 3. Results

Among the Brazilian participants, all the interviewed nurses were female (n = 15) and between 40 and 45 years old, with a time of training between 10 and 15 years. They had between 10 and 15 years of service in primary care. One of them had worked for 20 years in the same health service, since its creation.

Among the French participants, the majority were female (n = 13) between 30 and 50 years old, with a training time between 15 and 20 years. They had worked in PHC for between 1 and 5 years.

The interview with nurse N15-BRA revealed how the process of deinstitutionalization of a PHC model and the institutionalization of a new model has been occurring with the Health on the Hour Program in southern Brazil. Considering her eight years of management experience, nurse N15-BRA reported on the reduction in the number of professionals, which resulted in a diminished capacity to serve the population in this territory:

*When these two units [UBS + ESF] were merged, we had 70 h of clinical doctors for 60,000 thousand inhabitants... it was a huge demand... Everything we tried to implement in previous years, the nurse’s reception was ruined because when you open the door in the morning and there are 50 people waiting and in your agenda there are 20 appointments available... either it is Sofia’s choice or the person who arrived at 4:30 in the morning, even if she is not the most important one in the queue, do you have the courage to say that she is not going to get what she wants? In the end, the reception ended and inevitably became a form because... (…), so all the work we tried to implement in the previous years, we had enough nurses, we had enough technicians, went down the drain... when in 2013... the unit came with 20, with three nursing technicians, even though they are all 40 h.... (…), so there was a fabulous scrapping over the years since I entered, it comes in a huge scrapping... so it was a kind of torturing thing, and with the “Health on the Hour”, the order was the (educational) groups should be done by nursing technicians and community agents, that is, it is taking away from the nurses all the work of prevention and health promotion and... the goal at that moment... what was important was this: even if they want to say no, they get angry, the management. I say, they get angry with this speech; in fact it is a big trick. We became an ER, we became an Emergency Room. The order is 75% of consultations are spontaneous demand; that is, you don’t have anything scheduled*

(N15-BRA Health Center).

The term “torturous” and the metaphor of “Sophie’s choice” expressed the attempt to reorganize the reception of users of the health service, with the changes in the nurses’ practices introduced by management. Between “Sophie’s choice” and the order of arrival in line, the choice was for the order of arrival in line, and saying “no” to surplus users.

Although the manager avoided “Sophie’s choice,” she needed to make choices. Making choices and making dilemmatic decisions has been the daily life of managers and health care professionals in the face of the transformations introduced by the NPM. The metaphor “Sophie’s choice” is an expression used in reference to William Styron’s novel, published in 1979, in which the character is faced with a dilemma and she needs to make a decision between two situations considered antagonistic or incompatible, which in the case of the novel means choosing which one of her children will live.

The particularity presented in the Brazilian context seems not to be so particular, as there are resonances with the French context. In France, nurses from the Medical-Psychological Center (CMP) and the Center for Maternal and Child Protection (PMI) revealed convergences regarding the disinvestment in public health facilities in recent decades.

N-FRA explained that there was an increase in the number of users in this territory, as the need for mental health interventions had increased. Since there was no possibility of attending to all the patients, the surplus was referred to private facilities. N-FRA also explained that they could not meet the demand:

*And we also have... the CMP has... has been around since then, so I said since the 1970s, and at that time there was a certain number of inhabitants, and there was a CMP for a certain number of inhabitants, and since then there has been a lot more... more people living in the city and there is only one CMP, and normally it should create... open a second CMP, but that will be in the future... because there are many patients who need psychiatric care... so there are patients we cannot serve. (…) when the patient has means (purchasing power), I ask him to see a doctor... to see a liberal psychiatrist, to see a liberal nurse; we cannot take care of everybody*

(N14-FRA CMP).

*(...) since the other PMI doesn’t have a doctor, so we did the consultation here to put it there, in concrete terms. So, normally there were consultations on Mondays, Tuesdays, Wednesdays, and Thursdays... except that we split the time, we no longer fully meet the needs of the population, there is a lack of consultations, we do not meet all the demand we have... we meet the minimum*

(N12-FRA PMI).

Nurses N4-BRA and N5-BRA worked in health services that implemented the Health on the Hour program in southern Brazil, offering advanced access to PHC:

*(...) we work with advanced access, so we schedule from one day to the next... 70% of it is free demand, 30% scheduled. Today I have these three patients scheduled, but per shift our schedule is eight appointments/shift, which is half an hour (30 min). The doctor is 12, which is every 20 min*

(N5-BRA USF).

N4-BRA expressed her disagreement with this new model in the health care facility, as she related advanced practice nursing to the precarization of health care work and the overload of nursing work:

*(...) I think that each one has their own field of knowledge and performance. We are a team, we need physicians, not validating horrible protocols and putting us ahead of things... there are nurses who like and think that they don’t need physicians for anything. I have another thought; I disagree a lot with that. I think this is dangerous, you know. The nurse that likes it, he will like this power of having the prescription, but he doesn’t see that behind this, the work overload and also the precariousness, you know, we are a cheaper labor force than the doctor*.

(N4-BRA Street office).

On the other hand, nurse N1-BRA, working in the southeast region of Brazil, envisioned advanced practice as a new professional space, seeking social valorization, economic enhancement, and the acquisition of new competencies:

*Today I had a difficulty while you were waiting for me. It frustrates me, a thousand crosses, ah, I had to be a doctor, no, I had to be a nurse, for example, when we talk about advanced nursing practice of expanded nursing practice, we need to have protocols or change in legislation for this. I saw a woman today, who came here for a women’s health consultation, for cervical cytology (…) she demanded pharmacological treatment, which in places that have adequate protocols, nurses are prescribers. (…) I really can’t, and it is not because I don’t know how to solve it, I would know today what to prescribe for the patient, the dose, how the patient should use it, how to write the prescription, but in the municipality where I work, if the patient comes with a prescription stamped by me, but the nurse did this? Wow, I’ll end up in the Federal Council of Medicine to answer a lawsuit. (…) I had no way to solve the patient’s problem, autonomously*

(E1-BRA NSF–individual interview).

In France, the Maison de Santé (in English, House of Health) is a new format of health establishment that integrates the logic of the primary care typical of developed countries, a composition of curative and preventive activities. In an interview with the nurse of one of these services, N9-FRA presented the health project of this establishment, which prioritized the care of people with chronic diseases such as diabetes, cardiovascular problems, drug use, and childhood obesity:

*The health project includes patient therapeutic education for diabetes, hypertension, childhood obesity, chronic obstructive pulmonary disease (COPD) patients, smokers, vaccinations, cancer tests, and Pink October activities*

(N9-FRA Maison de Santé).

The protocols that the nurses used were also presented:

*The protocols followed are the testing and management of patients with high cardiovascular risk, plantar perforating malady, wound monitoring, oral anticoagulants, low back pain, and COPD. The team plans today the insulin therapy protocol*

(N9-FRA Maison de Santé).

The people who seek care at the Maison de Santé are seen in the health service, but they are not the main focus of the health project. This point sheds light on the differences between the French model and the Brazilian model. In Brazil, we have instituted ESF as the main model of reorientation of care practices in PHC, focusing on the family and the territory. In France, the Maison de Santé focuses on providing care to certain (children, women, the elderly) or people with specific pathologies with a higher incidence in the territory.

The liberal character of this model became even clearer during the first author’s participation in the virtual congress Avec les Équipes, promoted by the French Federation of Maisons de Santé Pluriprofessionnelles (FFMSP), in September 2021. The FFMSP is chaired by a physician, and much of the coordination of the tables at the event was conducted by physicians.

Discussions about the role of the Maisons de Santé and the Professional Territorial Health Communities (PTHC) were debated, revealing tensions and resistance regarding the participation of the Maisons de Santé in local health networks. There was a demand for greater openness on the part of the PTHC to the Maisons de Santé. The competitive character, the business logic, and the “seductive air” of the private sector strongly permeated the discussions in this meeting, in which individuals looked like businessmen competing for a slice of the market. Under the slogan “les précurseurs de l’avenir demain” (in English, the precursors of the future tomorrow), the physicians asked the representatives of the Regional Health Agency (RHA) present at the event: “soigner le précurseur demain, la solution vient du terrain, nous sommes pour les accompagner, nous libère d’un certain numéro de contraintes administratifs” (in English “take care of the precursor of tomorrow, the solution comes from the field, we are for the accompaniment, free us from a number of administrative restrictions”).

At Maison de Santé, nursing work involves preventive and curative activities, developed in a multidisciplinary team. However, the list of activities carried out confers less autonomy, compared to the Brazilian experience. An example is the Haemoglucotest (HGT), which cannot be performed without a medical prescription.

One of the highlights of the nursing event was awarding of a prize to the best videos submitted by the registered Maisons de Santé. The award was named Régine Langlade after a nurse from a Maison de Santé who died from COVID-19 in 2021.

## 4. Discussion

A fragment of speech of one of the Brazilian nurses expressed the authoritarian character with which the public health policy “Health on the Hour” crossed the professional practice and autonomy of PHC nurses, determining actions that should or should not be performed by these professionals in that health service. This demonstrates the way in which professional practices are crossed by policies and management logics. Research on public policies does not always take into account the fine analysis of the effects on professional practices, and how these practices impact the implementation of these policies.

The discussion about the crossing of state and/or party policies in professional practices in health appears in a study from the 1990s, with nurses and doctors from UBS in Ribeirão Preto, carried out during the period when the Brazilian state reforms were beginning [34]. This same study showed that public policies caused negative interference in professional practices, being considered external to their work because they were arbitrary, normative, vertical, distant from the practices, difficult to make feasible, elaborated by “politicians” and not technicians, and were seen as an electioneering practice [37].

The Health on the Hour program, according to the Ministry of Health, aims to expand access to PHC through, for example, extended hours of service in UBS [38]. However, there are important criticisms of this program, which indicate that it places too much value on individual and curative care, and mischaracterizes the ESF model and the logic of reorientation of the health care model, which has been considered an example of successful public health policy in force for 27 years in Brazil [39,40]. From the reformist governmental perspective, the Health on the Hour program and the other policies implemented as of 2019 present the premise of strengthening the attributes of PHC and organizational modernization in the 21st century.

Advanced nursing practices were mentioned in the interviews as a way out of situations in which there is a shortage of medical professionals in the health service, and as a way to expand user access to health services. One author defines three main reasons for adopting advanced practices: improved access, improvements in care, and cost reduction [41].

According to the same author, advanced practices have emerged in developed countries, such as Canada and the United States, in the form of master’s degree courses in which nurses develop expanded clinical competencies in user care [41]. Advanced nursing practices emerged from the shortage of health workers in certain areas at the same time that there was an increase in health care costs, leading health care systems to find alternatives for expanding access and universal coverage. Advanced nursing practices lead systems to seek greater efficiency, including delegating practices that were initially medical to nurses without impacting remuneration, which reinforces the aspect of NPM that prioritizes cost reduction.

Some authors envision greater autonomy in the role of advanced nursing practice, with the recognition of a title and with supervision by a physician (depending on the context), which may launch new directions for Latin America and the Caribbean with regard to universal access to health care and universal health coverage [42]. However, the speech of nurse E4-BRA, who had been experimenting with advanced access, expressed that the result had been work overload, not social and salary valorization. The issue of work overload seems to resonate with the results of an investigation about the implementation of advanced access in the municipality of Diadema in the state of São Paulo [43]. With the progressive reorganization of the schedules of physicians, nurses, and nursing technicians, 75% were assigned to spontaneous demand and 25% to scheduled demand. With the advanced access, an increase of 36% was identified in the number of medical appointments, a 792% increase in the number of nurse appointments, and 400 users were attended to by nursing technicians, which was previously null [43].

In the present investigation, advanced access acted as an institutional transducer of the NPM, acting as a useful tool for cost rationalization, in which the result produced was exactly the opposite of what it proposes. Instead of expanding access, it reduced it, and provoked effects on professional practices and on the way health care is produced.

Institutional transducers are “devices (agency of actions and relations) aimed at converting institutional processes into more integrative ones” [44] (p. 178). According to the author, a transducer is a device that transforms a flow from one unit of measurement into another unit; for example, a hydroelectric dam transforms hydraulic force into electric force [44]. As an institutional transducer, advanced access transforms professional nursing practices in primary care into standardized practices, with low autonomy and the valorization of a clinic based on individual consultations to the detriment of collective health education and promotion activities. Quantity and percentages are the references for evaluating the quality of service.

A systematic review entitled “Nurses as substitutes for physicians in primary care”, which analyzed 18 studies from high-income countries, showed that nurses have longer appointments and patients are more likely to maintain follow-up [45]. The study also revealed greater satisfaction with care, with patients having similar or better outcomes in health areas such as heart disease, diabetes, and rheumatism, among others. The review concluded that the impact on costs of care were uncertain, with no difference in the number of prescriptions between nurses and physicians [45].

In France, the institutionalization of advanced nursing practice is recent, being authorized by the Health System Modernization Act in 2016 [46]. In France, there are 28 universities that offer master’s degrees courses lasting from two to three years. It is estimated that 3% to 5% of French nurses have the title of Infirmière de Pratique Avancée (IPA), according to data from the French National Association of Nurses in Advanced Practice [46]. The title is awarded to five areas: (1) stabilized chronic pathologies–prevention and current polypathologies in primary care; (2) oncology and onco-hematology; (3) renal failure, dialysis, and transplantation; (4) psychiatry and mental health; and (5) emergencies [43]. Being called “super-nurses”, these nurses act in the following domains of intervention: (a) orientation, education, prevention, and testing; (b) conclusive clinical evaluation; and (c) prescription of complementary tests and the renewal or adaptation of prescriptions. However, it is the physician who determines the domain of intervention and the patients who will be proposed for follow-up by a nurse in advanced practice [46].

The creation of a Maison de Santé must present inclusion and exclusion criteria for the project. Unlike the Brazilian model, it is not family-oriented. To create a new Maison de Santé, a health project must be submitted to the calls for proposals regularly published by the RHA. These projects must follow the criteria outlined in the city’s policies for the priority neighborhoods.

It should be noted that there is great variability in the constitution of the teams of the Maisons de Santé, reflecting a type of selective PHC, which establishes a minimum basket of services offered to the population. In developed countries, PHC is the first level of health care, composed of a set of outpatient services of first contact integrated into a public system of universal access [47]. Debates about the institutionalization of these public–private partnerships is common in France as well, and criticism that arises is linked to a number of issues, such as the nature and effectiveness of cooperation between actors, the power relations that develop, the resource asymmetries that persist, and the consequences of the increasing and formalized participation of health cooperation companies [48].

In France, discussion about transformations in the public’s mission is not new; the arguments are to make its mission evolve over time, according to social needs and technological changes:

(...) the implicit philosophy is that there is a difference between being responsible for an activity and doing it yourself. The state can be responsible and the activity performed or implemented by a private body. The idea is not new in French law, but the techniques are. Does the state necessarily have to be an owner or employer to provide services to citizens, or can it buy services from the best providers? This is how the question of reviewing missions in OECD countries is posed [49] (p. 3).

## 5. Conclusions

Professional nursing practices in PHC have been strongly pressured by new management practices, leading to work precarization and staff reduction. The results showed that making dilemmatic decisions has been the daily routine of nurses, and the NPM has not resulted in debureaucratization and higher quality of care. Some health policies that have been implemented in a set of reforms in primary care have acted as institutional transducers; that is, instead of expanding user access to health services, they have caused the opposite, namely, a reduction in access.

As for the contributions to the practices of nurses in PHC, the research showed that the changes underway in management may not represent advances in the profession, such as social and salary valorization. On the contrary, the result has been precariousness, work overload, and even change of profession in the case of France.

The research results contribute to the understanding of the modes of implementation of health policies and management. It demonstrates how these policies and management cut across professional practices and how they are conceived, transformed, and implemented by health professionals. The results of this study also show that the ongoing reforms, based on cost reduction, have been reducing the supply of public health actions and services, referring surplus users to private services.

The study points out that the global perspectives of management in health systems, as is the case of New Public Management, strongly interfere with the work of nurses, and therefore impact the workforce and access and health care.

Among the limitations of the study are the non-inclusion of nursing technicians and assistants in Brazil, nursing assistants (aide-soigants) in France, and other professional categories in the health area, which could bring more elements for analysis and could aid in understanding some of the tensions, such as those that occur within the medical profession.

## Data Availability

The data presented in this study are available on request from the corresponding author. The data are not publicly available due to confidentiality and privacy concerns.

## References

[1] Paula A.P.P. (2014). Por uma Nova Gestão Pública.

[2] Dasso Júnior A.E. (2016). “Nova Gestão Pública” (NGP): A teoria de administração pública do estado ultraliberal. Proceedings of the XXIII Encontro Nacional do Conselho Nacional de Pesquisa e Pós-Graduação em Direito no Brasil (CONPEDI).

[3] Bresser-Pereira L.C. (2017). Reforma gerencial e legitimação do estado social. Rev. Adm. Publica.

[4] Pesqueux Y. (2020). New Public Management (NPM) et Nouvelle Gestion Publique (NGP). Doctoral Thesis.

[5] Soares L.T., Giovanella L., Escorel S., Lobato L.V.C., Noronha J.C., Carvalho A.I. (2017). Políticas sociais na América Latina. Políticas e Sistema de Saúde No Brasil.

[6] Pierru F. (2022). “La Grande Sécu”: Une réorientation substantielle du financement de la santé en France?. Raison Présente.

[7] Chiavegato Filho L.G., Navarro L.V. (2014). A ideologia gerencialista no Sistema Único de Saúde (SUS): A organização do trabalho de médicos. Psicol. Rev..

[8] Jamra C.C.A., Cecílio L.C.D.O., Correia T. (2021). Os médicos e a racionalização das práticas hospitalares: Novos limites para a liberdade profissional?. Saúde Debate.

[9] Terra L.S.V., Campos G.W.D.S. (2019). Alienação do trabalho médico: Tensões sobre o modelo biomédico e o gerencialismo na atenção primária. Trab. Edu. Saúde.

[10] Bezes P., Chiapello E., Desmarez P. (2016). La tension savoirs-pouvoirs à l’épreuve du gouvernement par les indicateurs de performance. Sociol. Du Trav..

[11] Gay B. (2013). Repenser la place des soins de santé primaires en France-Le rôle de la médecine générale. Rev. D’épidémiologie St. Publique.

[12] Moyal A. (2019). Rationalisation des pratiques professionnelles en maisons de santé pluriprofessionnelles. Rev. Française Sci. Polit..

[13] Simonet D. (2013). New public management and the reform of French public hospitals. J. Public Aff..

[14] Resenterra F., Siggen M., Giauque D. (2013). Les cadres intermédiaires entre contraintes managériales et défense des identités professionnelles: L’exemple des Hôpitaux de Suisse Romande. Humanisme Entrep..

[15] Hoyle L. (2014). Nurses’ perception of senior managers at the front line: People working with clipboards. J. Adv. Nurs..

[16] Bergh A.L., Friberg F., Persson E., Dahlborg-Lykhage E. (2015). Perpetuating “New Public Management” at the expense of nurses” patient education: A discourse analysis. Nurs. Inq..

[17] Winblad U., Blomqvist P., Karlsson A. (2017). Do public nursing home care providers deliver higher quality than private providers? Evidence from Sweden. BMC Health Serv. Res..

[18] Tingvoll W.A., Sæterstrand T., Mcclusky L.M. (2016). The challenges of primary health care nurse leaders in the wake of New Health Care Reform in Norway. BMC Nurs..

[19] Henderson J., Willis E., Toffoli L., Hamilton P., Blackman I. (2016). The impact of rationing of health resources on capacity of Australian public sector nurses to deliver nursing care after-hours: A qualitative study. Nurs. Inq..

[20] Willis E., Toffoli L., Henderson J., Couzne L., Hamilton P., Verrall C., Blackman I. (2016). Rounding, work intensification and new public management. Nurs. Inq..

[21] Willis E., Carryer J., Harvey C., Pearson M., Henderson J. (2017). Austerity, new public management and missed nursing care in Australia and New Zealand. J. Adv. Nurs..

[22] Cooper D., Mercer D. (2017). Professional and political reflections on the “grand round”: A critical case study. Br. J. Nurs..

[23] Bogeskov B.O., Rasmussen L.D., Weinreich E. (2017). Between meaning and duty–leaders’uses and misuses of ethical arguments in generating engagement. J. Nurs. Manag..

[24] Berlogey N. (2018). Nova Gestão Pública e profissões hospitalares. Saúde Soc..

[25] Sebai J., Yatim F. (2018). Approche centrée sur le patient et Nouvelle Gestion Publique: Confluence et paradoxe. St. Publique.

[26] Rivière A. (2019). Tensions de rôle et stress professionnel chez les cadres de santé à l’hôpital public: L’effet modérateur des stratégies d’ajustement. Rev. Française Gest..

[27] Drolet M.J., Lalancette M., Caty M.E. (2020). «Brisées par leur travail! OU Au bout du rouleau»: Réflexion critique sur les modes managériaux en santé. Rev. Can. Bioéthique.

[28] Roque H., Veloso A., Silva I., Costa P. (2015). Estresse ocupacional e satisfação dos usuários com os cuidados de saúde primários em Portugal. Cien Saude Colet..

[29] Harzheim E. (2020). “Previne Brasil”: Bases da reforma da Atenção Primária à Saúde. Ciên. Saúde Colet..

[30] Lourau R. (2014). A Análise Institucional.

[31] Monceau G., Fortuna C.M. (2020). Le pouvoir des sujets dans les institutions: De sa négation à sa reconnaissance. Nouv. Rev. Psychosociologie.

[32] Monceau G., L’abbate S., Mourão L.C., Pezzato L.M. (2013). A socioclínica institucional para pesquisas em educação e em saúde. Análise Institucional e Saúde Coletiva.

[33] Fortuna C.M., Silva S.S., Mesquita L.P., Matumoto S., Oliveira P.S., Santana F.R. (2017). The institutional socio-clinic as a theoretical and methodological framework for nursing and health research. Texto Contexto Enferm..

[34] Tong A., Sainsbury P., Craig J. (2007). Consolidated criteria for reporting qualitative research (COREQ): A 32- item checklist for interviews and focus group. Int. J. Qual. Health Care.

[35] Kasper M.S. (2022). Repercussões da Nova Gestão Pública nas Práticas de Enfermeiros na Atenção Básica no Brasil e nos Cuidados Primários na França. Doctoral Thesis.

[36] Paillé P., Mucchielli A. (2012). L’Analyse Qualitative en Sciences Humaines et Sociales.

[37] Almeida M.C.P. (1997). A enfermagem e as políticas de saúde. Esc. Anna Nery Rev. Enferm..

[38] Ministério da Saúde (2019). Portaria nº 930, de 15 de maio de 2019: Institui o Programa “Saúde na Hora”, que Dispõe Sobre o Horário Estendido de Funcionamento das Unidades de Saúde da Família.

[39] Giovanella L., Franco C.M., Almeida P.F. (2020). Política Nacional de Atenção Básica: Para onde vamos?. Ciência Saúde Coletiva.

[40] Massuda A. (2020). Mudanças no financiamento da Atenção Primária à Saúde no Sistema de Saúde Brasileiro: Avanço ou retrocesso?. Ciência Saúde Coletiva.

[41] Toso B.R.G.O. (2016). Práticas avançadas de enfermagem em atenção primária: Estratégias para implantação no Brasil. Enferm. Foco..

[42] Cassiani S.H.B., Silva F.A.M. (2019). Expanding the role of nurses in primary health care: The case of Brazil. Rev. Lat. Am. Enferm..

[43] Cirino F.M.S.B., Schneider Filho D.A., Nichiata L.Y.I., Fracoll L.A. (2020). O Acesso Avançado como estratégia de organização da agenda e de ampliação do acesso em uma Unidade Básica de Saúde de Estratégia Saúde da Família, município de Diadema, São Paulo. Rev. Bras. Med. Família E Comunidade.

[44] Monceau G. (2009). Socio-Clinique Institutionnelle et Education. Parcours, Théorisations et Méthodologie.

[45] Laurant M., Biezen M., Wijers N., Watananirun K., Kontopantelis E., Vught A.J. (2018). Nurses as substitutes for doctors in primary care. Cochrane Database Syst. Rev..

[46] Association Nationale Française des Infirmières en Pratique Avancée. https://anfipa.fr/colleges.html.php.

[47] Giovanella L., Mendonça M.H.M., Giovanella L., Escorel S., Lobato L.V.C., Noronha J.C., Carvalho A.I. (2017). Atenção Primária à Saúde. Políticas e Sistema de Saúde No Brasil.

[48] Guilbaud A. (2015). Les partenariats public-privé sanitaires internationaux: Diffusion et incarnation d’une norme de coopération. Mondes Dev..

[49] Trosa S. (2007). Le public et le privé: La révision des missions débouche-t-elle sur la sous-traitance? De l’essence à l’existence. Polit. Et Manag. Public.

